# Genome Sequence of Beauveria bassiana Strain ATCC 74040, a Widely Employed Insect Pathogen

**DOI:** 10.1128/MRA.00446-20

**Published:** 2020-06-04

**Authors:** Rossano Atzeni, Gabriele Moro, Maria Giovanna Marche, Paolo Uva, Luca Ruiu

**Affiliations:** a CRS4, Pula, Italy; b Bioecopest Srl, Alghero, Italy; c Dipartimento di Agraria, University of Sassari, Sassari, Italy; Vanderbilt University

## Abstract

The broad-spectrum insecticidal activity of Beauveria bassiana strain ATCC 74040 is well documented. The whole-genome sequence of this strain is reported here, revealing a plethora of genes related to its insecticidal potential and providing new insights on the mechanism of action.

## ANNOUNCEMENT

Beauveria bassiana (Bals.) Vuill. (Ascomycota, Hypocreales) is a naturally occurring entomopathogenic fungus, representing one of the first applications of insect pest microbial control in the 19th century (1). A few decades of research dedicated to this species led to the isolation of several strains showing different levels of virulence against different targets. The implication of specific genes in the pathogenicity process has been demonstrated, but many aspects of the biology of this species, including its complex insecticidal mechanism of action, are not yet fully understood (2, 3). The recent whole-genome sequencing and annotation of strain ARSEF 2860 provided an evolutionary framework and an overview of the genetic traits related to the pathogenicity and virulence of the fungus (4). However, genomic differences between strains are expected (5).

B. bassiana strain ATCC 74040 (ARSEF 3097) was originally isolated from a boll weevil cadaver in the Rio Grande Valley of Texas (6) and has been safely used commercially in biological pest management for a few decades. In addition to significant insecticidal action against a variety of crop pests through contact, endophytic properties of this strain have recently been demonstrated, highlighting its potential against piercing-sucking insects (7). Sequencing, assembly, and annotation of the whole B. bassiana ATCC 74040 genome provide valuable information on its specific arsenal of insecticidal toxins and virulence factors.

The B. bassiana ATCC 74040 strain used for sequencing was isolated from the commercial product Naturalis (CBC [Europe] Srl) and grown on Sabouraud dextrose agar to isolate genomic DNA employing the DNeasy blood and tissue kit (Qiagen GmbH, Hilden, Germany) (8). After being quantified using a Qubit 2.0 fluorometer (Life Technologies, Carlsbad, CA, USA), DNA was supplied to the next-generation sequencing facilities of Porto Conte Ricerche Srl (Alghero, Italy). The sequencing libraries were prepared according to the Nextera XT DNA sample preparation protocol (Illumina, San Diego, CA, USA). Pooling and clustering of libraries were performed employing the Illumina cBot system, in agreement with the Illumina TruSeq paired-end cluster kit protocol. DNA was sequenced using an Illumina HiScanSQ sequencer, according to the paired-end approach. Whole-genome sequencing resulted in 48,744,209 paired-end reads with a length of 93 bp, which were trimmed and quality filtered with Trim Galore v0.6.4 and FastQC v0.11.8 (9), respectively. SPAdes v3.14.0 (10) was used with the “careful” option for *de novo* assembly, while QUAST v5.0.2 (11) was used for genome assembly evaluation. Default parameters were used except where otherwise noted.

Based on contigs of ≥500 bp, the draft genome sequence obtained contains 2,322 contigs, with an *N*_50_ value of 36,979 bp, a GC content of 51.86%, and a total estimated size of 32,401,214 bp. The genome was annotated using GlimmerHMM v3.0.4 (12), which led to the identification of 20,271 predicted genes, including genes related to the insecticidal potential, such as chitinases, proteases, and hydrophobins. Preliminary information on the B. bassiana strain ATCC 74040 virulence specificity was gained by aligning the quality-filtered reads to the B. bassiana ARSEF 2860 reference genome (GenBank accession number ASM28067v1). Further comparative studies are needed to investigate the genomic variability within this entomopathogenic species.

### Data availability.

The complete genome sequence of Beauveria bassiana ATCC 74040 (=ARSEF 3097) is available in DDBJ/ENA/GenBank under the accession number JABAOI000000000. The raw reads have been deposited in the NCBI database under the SRA number SRR11521919.

## References

[1] Ruiu L 2018 Microbial biopesticides in agroecosystems. Agronomy 8:235. doi:10.3390/agronomy8110235.

[2] Ortiz-Urquiza A, Keyhani NO 2016 Molecular genetics of *Beauveria bassiana* infection of insects. Adv Genet 94:165–249. doi:10.1016/bs.adgen.2015.11.003. 27131326

[3] Valero-Jiménez CA, Wiegers H, Zwaan BJ, Koenraadt CJM, van Kan J 2016 Genes involved in virulence of the entomopathogenic fungus *Beauveria bassiana*. J Invertebr Pathol 133:41–49. doi:10.1016/j.jip.2015.11.011. 26628209

[4] Xiao G, Ying S-H, Zheng P, Wang Z-L, Zhang S, Xie X-Q, Shang Y, Leger RJ, St Zhao G-P, Wang C, Feng M-G 2012 Genomic perspectives on the evolution of fungal entomopathogenicity in *Beauveria bassiana*. Sci Rep 2:483. doi:10.1038/srep00483. 22761991 PMC3387728

[5] Valero-Jiménez CA, Faino L, Spring In't Veld D, Smit S, Zwaan BJ, van Kan JAL 2016 Comparative genomics of *Beauveria bassiana*: uncovering signatures of virulence against mosquitoes. BMC Genomics 17:986. doi:10.1186/s12864-016-3339-1. 27905873 PMC5134283

[6] Wright JC 14 May 1996 Biological ovicide for control of lepidopterous insects. US Patent 5516513.

[7] Rondot Y, Reineke A 2018 Endophytic *Beauveria bassiana* in grapevine *Vitis vinifera* (L.) reduces infestation with piercing-sucking insects. Biol Control 116:82–89. doi:10.1016/j.biocontrol.2016.10.006.

[8] Falchi G, Marche MG, Mura ME, Ruiu L 2015 Hydrophobins from aerial conidia of *Beauveria bassiana* interfere with *Ceratitis capitata* oviposition behavior. Biol Control 81:37–43. doi:10.1016/j.biocontrol.2014.11.005.

[9] Andrews S 2010 FastQC: a quality control tool for high throughput sequence data. Babraham Bioinformatics, Babraham Institute, Cambridge, United Kingdom http://www.bioinformatics.babraham.ac.uk/projects/fastqc.

[10] Bankevich A, Nurk S, Antipov D, Gurevich AA, Dvorkin M, Kulikov AS, Lesin VM, Nikolenko SI, Pham S, Prjibelski AD, Pyshkin AV, Sirotkin AV, Vyahhi N, Tesler G, Alekseyev MA, Pevzner PA 2012 SPAdes: a new genome assembly algorithm and its applications to single-cell sequencing. J Comput Biol 19:455–477. doi:10.1089/cmb.2012.0021. 22506599 PMC3342519

[11] Gurevich A, Saveliev V, Vyahhi N, Tesler G 2013 QUAST: quality assessment tool for genome assemblies. Bioinformatics 29:1072–1075. doi:10.1093/bioinformatics/btt086. 23422339 PMC3624806

[12] Majoros WH, Pertea M, Salzberg SL 2004 TigrScan and GlimmerHMM: two open source ab initio eukaryotic gene-finders. Bioinformatics 20:2878–2879. doi:10.1093/bioinformatics/bth315. 15145805

